# A New Coarse Gating Strategy Driven Multidimensional Assignment for Two-Stage MHT of Bearings-Only Multisensor-Multitarget Tracking

**DOI:** 10.3390/s22051802

**Published:** 2022-02-24

**Authors:** Zheng Wei, Zhansheng Duan, Yina Han, Mahendra Mallick

**Affiliations:** 1 Center for Information Engineering Science Research, Xi’an Jiaotong University, Xi’an 710049, China; weizheng179@stu.xjtu.edu.cn; 2 School of Marine Science and Technology, Northwestern Polytechnical University, Xi’an 710072, China; yina.han@nwpu.edu.cn; 3 Independent Researcher, Anacortes, WA 98221, USA; mmallick.us@gmail.com

**Keywords:** bearings-only multisensor-multitarget tracking, multidimensional assignment (MDA), coarse gating, Mahalanobis distance, maximum likelihood estimation, multiple hypothesis tracking

## Abstract

The problem of two-dimensional bearings-only multisensor-multitarget tracking is addressed in this work. For this type of target tracking problem, the multidimensional assignment (MDA) is crucial for identifying measurements originating from the same targets. However, the computation of the assignment cost of all possible associations is extremely high. To reduce the computational complexity of MDA, a new coarse gating strategy is proposed. This is realized by comparing the Mahalanobis distance between the current estimate and initial estimate in an iterative process for the maximum likelihood estimation of the target position with a certain threshold to eliminate potential infeasible associations. When the Mahalanobis distance is less than the threshold, the iteration will exit in advance so as to avoid the expensive computational costs caused by invalid iteration. Furthermore, the proposed strategy is combined with the two-stage multiple hypothesis tracking framework for bearings-only multisensor-multitarget tracking. Numerical experimental results verify its effectiveness.

## 1. Introduction

Multitarget tracking (MTT) refers to jointly estimating the number of targets and their states in the presence of false alarms and missed detections using single or multiple sensors [1]. It has been widely used in many fields such as surveillance and tracking of ground moving targets [2], maritime surveillance [3], sonar tracking of submarines [4], simultaneous localization and mapping [5], unmanned air vehicles [6], etc. For different application scenarios, tracked targets can be considered as point targets or extended targets [7]. If the distance between the sensor and target is large enough as in radar-based air surveillance applications, the target can be treated as a point target. In this case, it is usually assumed that a target can give rise to at most one measurement in a scan [8]. However, if multiple resolution cells of the sensor are occupied by a target, for example, in vehicle tracking using automotive radar, the target is regarded as an extended target [9]. In such a case, each target can give rise to multiple measurements [10]. Only point targets will be discussed below.

Multitarget tracking has been studied for decades and many effective algorithms are available. The earliest and simplest MTT algorithm is the global nearest neighbor (GNN) algorithm [11], which attempts to search for the single most likely hypothesis for track update and new track initiation [12]. Although the GNN algorithm is intuitively attractive and easy to implement, it is prone to track loss in scenarios with closely spaced targets and high false alarm density [13]. The joint probabilistic data association (JPDA) algorithm is an extension of the probabilistic data association (PDA) algorithm to the multitarget case [14]. The standard JPDA algorithm evaluates the association probabilities of measurement-to-track and combines them to obtain the state estimate of the target [15], which means that one observation may contribute to updating multiple tracks [16]. Many variants of the JPDA algorithm are abundant, such as the joint integrated PDA (JIPDA) algorithm [17] and multiscan JPDA (MS-JPDA) algorithm [18]. Multiple hypothesis tracking (MHT) is a deferred decision algorithm for MTT. It handles uncertainty of measurement-to-track associations by considering all possible association hypotheses in subsequent multiple scans [19]. Compared with GNN and JPDA algorithms that rely on the current scan, the MHT algorithm is computationally expensive, but it has significantly better tracking performance [20]. There are two different implementations of MHT algorithm, namely hypothesis-oriented MHT [21] and track-oriented MHT [22]. Between them, the track-oriented MHT algorithm, which uses the score function to evaluate the quality of tracks, is considered a more effective alternative to a hypothesis-oriented MHT [21]. Among the above three data association-based MTT algorithms, i.e., GNN, JPDA, and MHT, MHT is considered as a leading algorithm in high false alarm density and dense target scenarios [23].

The random finite set (RFS) algorithm [24] represents the multitarget state and measurements as a random finite set, which allows multitarget tracking to be cast in a Bayesian framework to obtain an optimal multitarget Bayes filter. Due to the high computational complexity of a multitarget Bayes filter [25], many approximate filters have been developed, such as probability hypothesis density (PHD) [26], cardinalized PHD (CPHD) [27], second-order PHD [28], and multitarget multi-Bernoulli (MeMBer) [29] filters. It should be note that none of these filters can obtain distinguishable target tracks. The generalized labeled multi-Bernoulli (GLMB) [20] is the RFS based MTT algorithm that produces tracks. In recent years, the GLMB filter has been widely studied, and fruitful achievements have been achieved in both theory and application [30]. In addition, the GLMB filter has been used to develop an MTT algorithm with structures similar to MHT [19].

Multisensor-multitarget tracking (MSMTT) has two basic architectures: centralized and distributed tracking [7]. In centralized MSMTT, the raw measurements from all sensors are sent to the fusion center (FC) where data association is followed by filtering, while in distributed MSMTT, each sensor first processes its own measurements and then sends the results to FC for further processing. Both frameworks have their own advantages and disadvantages in terms of communication requirements, computational complexity, performance, robustness, etc. In general, the centralized MSMTT framework has higher accuracy [31]. However, in practical applications, due to network bandwidth limitations, it is often not feasible to communicate all measurements to FC. Comparatively, the distributed MSMTT framework can reduce communication cost and has better flexibility and reliability, but it is more challenging.

For distributed MSMTT based on data association, one approach is that each sensor sends the local track estimates to the FC, which performs track-to-track association and fusion [32]. Another type of approach is to perform measurement space tracking at individual local sensors to suppress clutter and then send the associated measurements to the FC where the measurement-to-track association is performed [33]. In addition, distributed MSMTT based on RFS has also been widely studied in recent years [34].

Depending on the types of sensors used, target tracking can be split into two classes: active and passive tracking [35]. The sensors used for active tracking first transmit signals (such as acoustic waves, electromagnetic waves) into the environment and then obtains range, bearing, elevation, and other measurements of the target of interest from the received echo [36]. Passive sensors sense the signal from the target of interest to acquire bearing, elevation, and other measurements. In comparison, passive tracking has the advantages of strong anti-interference and good concealment [37].

Passive tracking also involves a unique set of challenges. One of the key challenges in bearings-only tracking is that the range between the passive sensor and the target is unavailable. This results in an unobservability of the target state [38]. A basic observable condition is that the sensor performs a higher order maneuver than all targets [39]. An alternative approach is to use multiple spatially separated sensors for triangulation, that is, the passive MSMTT [40]. But for this approach, the attendant problem is the well-known ghosting. In order to reduce the number of ghosts, three or more sensors should be used [41]. In this case, multidimensional assignment (MDA) can be used to associate the measurements from different sensors to identify common targets, which also makes this approach computationally costly for a large number of measurements. One of the main reasons is that in MDA, most of the time (at least up to 80%), is spent in calculating the association cost [42]. To reduce calculation times, many fast MDA methods have been proposed. Among them, it was proposed in [43] to cluster the measurements of different sensors before forming possible association hypotheses, thus reducing the requirement for calculating the association cost. In addition, two improved MDA methods using prior track information were proposed in [44].

A new coarse gating strategy is studied for the passive MSMTT. First, in order to reduce the computational complexity of MDA, a new coarse gating strategy is proposed. Second, the proposed strategy is combined with a two-stage MHT (TS-MHT) framework for distributed MSMTT. The remainder of the paper is organized as follows. Section 2 formulates the problems of bearings-only MSMTT. Section 3 briefly summarizes MDA for measurement-to-measurement association. In Section 4, a new coarse gating is proposed. Section 5 presents the combination of the proposed new coarse gating driven MDA with the TS-MHT framework. Section 6 provides numerical examples to illustrate the effectiveness of the proposed coarse gating strategy. Section 7 concludes the paper.

## 2. Problem Formulation and Notations

**Figure 1:**
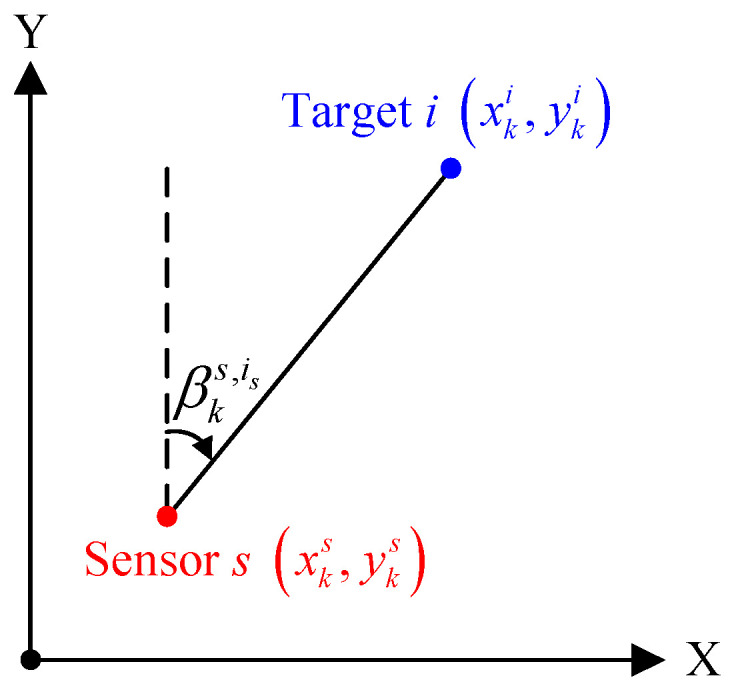
Illustration of two-dimensional bearing measurement.

The two-dimensional (2D) bearings-only MSMTT is considered. The bearing measurement is shown in Figure 1.

Assume that there are *S* synchronous passive sensors and sensor *s*, s∈{1,2,⋯,S}, can acquire $N_{s}$ bearing measurements ${\left\{z_{k}^{s,j_{s}}\right\}}_{j_{s}=1}^{N_{s}}$ at time *k*. Here, $N_{s}$ may not be equal to the number of true targets due to false alarms and nonunity detection probability $P_{D_{s}}$ of sensor *s*. For the sake of simplicity, each target is assumed to move with nearly constant velocity (NCV) in the XY-plane. Then, the discrete-time dynamic system can be written as follows:

(1) $$x_{k}^{i}=F_{k-1}x_{k-1}^{i}+w_{k-1}^{i},$$

(2) $$z_{k}^{s,j_{s}}=\left\{\begin{array}{cc} h\left(x_{k}^{i},p_{k}^{s}\right)+v_{k}^{s,j_{s}} & \mathrm{if}\;z_{k}^{s,j_{s}}\;\mathrm{originates}\;\mathrm{from}\;\mathrm{target}\;i\\ {\tilde{z}}_{k}^{j_{s}} & \;\mathrm{otherwise}\; \end{array}\right.,$$

where $x_{k}^{i}$ is the state vector consisting of the target position ${\left[x_{k}^{i}\;y_{k}^{i}\;\right]}^{\prime }$ and velocity ${\left[{\dot{x}}_{k}^{i}\;{\dot{y}}_{k}^{i}\right]}^{\prime }$, i.e., $x_{k}^{i}={\left[x_{k}^{i}\;{\dot{x}}_{k}^{i}\;y_{k}^{i}\;{\dot{y}}_{k}^{i}\right]}^{\prime }$, $F_{k-1}$ is the state transition matrix for NCV motion model, $\langle w_{k-1}^{i}\rangle$ is a sequence of zero-mean white Gaussian process noise, $p_{k}^{s}={\left[x_{k}^{s}\;y_{k}^{s}\right]}^{\prime }$ is the position of the sensor *s*, $\langle v_{k}^{s,j_{s}}\rangle$ is a sequence of zero-mean white Gaussian bearing measurement noise with variance $\sigma_{s}^{2}$, and the measurement noises across sensors are independent; *h* is a nonlinear function. The nonlinear relationship among $\beta_{k}^{i_{s}}$, $x_{k}^{i}$ and $p_{k}^{s}$ is given by the following:

(3) $$\beta_{k}^{s,i_{s}}=h\left(x_{k}^{i},p_{k}^{s}\right)=\tan^{-1}\left(x_{k}^{i}-x_{k}^{s},y_{k}^{i}-y_{k}^{s}\right),$$

where $\tan^{-1}$ refers to the four-quadrant inverse tangent function [45].

The purpose is to estimate the number of targets and their corresponding states in real time. A list of nomenclatures is provided in Nomenclatures.

## 3. Measurement-to-Measurement Association

**Figure 2:**
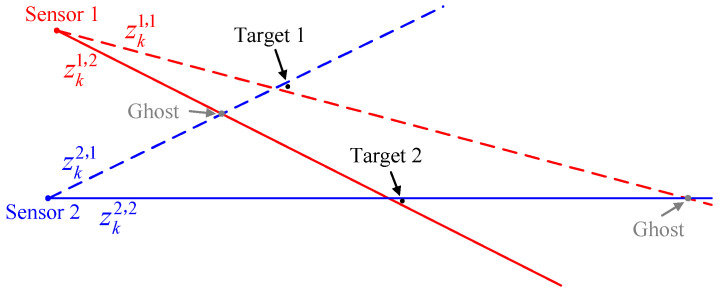
A scenario with 2 passive sensors and 2 targets.

A brief description of measurement-to-measurement association is required to illustrate the proposed strategy more clearly. For a single passive sensor, the range measurement between target and sensor is not available, which makes the target state unobservable. During target tracking, especially for track initiation, at least two passive sensors are needed to obtain the full position of the potential target. It should be noted that, in a two-dimensional multitarget tracking scenario with only two sensors, one of the major problems is the occurrence of false intersections or ghosts. For example, as shown in Figure 2, the dashed lines of different colors indicate bearing measurements originating from target 1, and the solid lines of different colors indicate bearing measurements originating from target 2. Obviously, the correct association pair cannot be identified with only two bearings-only sensors.

**Figure 3:**
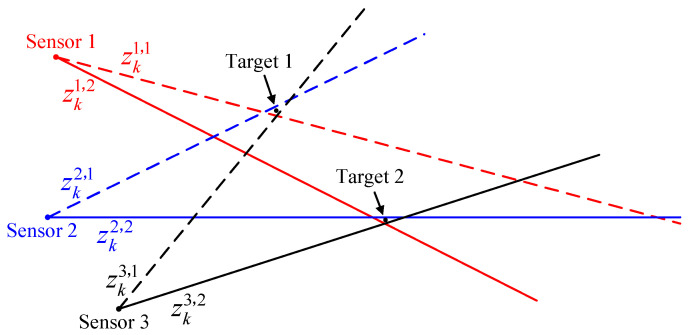
A scenario with 3 passive sensors and 2 targets.

Therefore, it is necessary to use three or more sensors if possible. However, the consequent problem is that this also makes it computationally expensive for a large number of measurements. Taking Figure 3 as an example, it shows the situation of two targets observed by three passive sensors with measurement errors.

**Figure 4:**
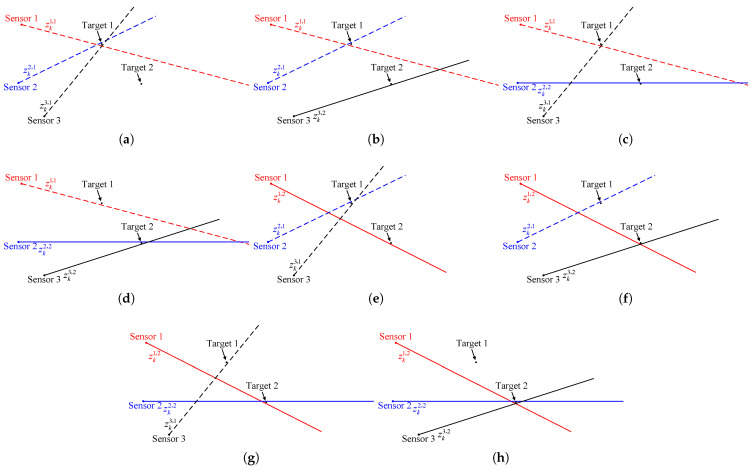
Geometric relationship between sensors and S-tuples of measurements (**a**) $Z_{k}^{111}$. (**b**) $Z_{k}^{112}$. (**c**) $Z_{k}^{121}$. (**d**) $Z_{k}^{122}$. (**e**) $Z_{k}^{211}$. (**f**) $Z_{k}^{212}$. (**g**) $Z_{k}^{221}$. (**h**) $Z_{k}^{222}$.

As shown in the above figure, the sets of measurements obtained by different sensors originating from the targets can be denoted by $\left\{z_{k}^{1,1},z_{k}^{1,2}\right\}$, $\left\{z_{k}^{2,1},z_{k}^{2,2}\right\}$, and $\left\{z_{k}^{3,1},z_{k}^{3,2}\right\}$, respectively. For measurement-to-measurement associations, each candidate association, consisting one measurement from each sensor, is denoted as the S-tuple of measurements $Z_{k}^{j_{1}j_{2}j_{3}}$. Even in the case where there are no false alarms or missed detections, the number of S-tuples is as follows:

(4) $$c=\left(\begin{array}{c} 2\\ 1 \end{array}\right)\left(\begin{array}{c} 2\\ 1 \end{array}\right)\left(\begin{array}{c} 2\\ 1 \end{array}\right)=2\times 2\times 2=8,$$

where $\left(\begin{array}{c} m\\ n \end{array}\right)$ denotes the number of combinations of selecting n choices from m choices. The corresponding geometric relationship is shown in Figure 4.

Each S-tuple of measurements is an association hypothesis. Obviously, only S-tuples $Z_{k}^{111}$ and $Z_{k}^{222}$ (as in Figure 4a,h) originate from the targets, and the others are spurious association hypotheses. Note that when there are false alarms or missed detections, and the number of S-tuples that can be formed will increase.

The process of associating the S-tuples of measurements to targets is the well-known measurement-to-measurement association problem. MDA based on likelihood ratio is widely considered to be the most efficient method to deal with this problem, which formulates the association between measurements from different sensors as a discrete optimization problem given by the following:

(5) $$\min_{\rho_{k}^{j_{1}j_{2}\cdots j_{S}}}\sum_{j_{1}=0}^{N_{1}}\sum_{j_{2}=0}^{N_{2}}\cdots \sum_{j_{S}=0}^{N_{S}}c_{k}^{j_{1}j_{2}\cdots j_{S}}\rho_{k}^{j_{1}j_{2}\cdots j_{S}}$$

subject to

(6) $$\begin{array}{c} \sum_{j_{2}=0}^{N_{2}}\sum_{j_{3}=0}^{N_{3}}\cdots \sum_{j_{S}=0}^{N_{S}}\rho_{k}^{j_{1}j_{2}\cdots j_{S}}=1,\;j_{1}=1,2,\cdots ,N_{1}\\ \sum_{j_{1}=0}^{N_{1}}\sum_{j_{3}=0}^{N_{3}}\cdots \sum_{j_{S}=0}^{N_{S}}\rho_{k}^{j_{1}j_{2}\cdots j_{S}}=1,\;j_{2}=1,2,\cdots ,N_{2}\\ \vdots \\ \sum_{j_{1}=0}^{N_{1}}\sum_{j_{2}=0}^{N_{2}}\cdots \sum_{j_{S-1}=0}^{N_{S-1}}\rho_{k}^{j_{1}j_{2}\cdots j_{S}}=1,\;j_{S}=1,2,\cdots ,N_{S} \end{array}$$

where $j_{s}=0$ is the index of dummy measurement to indicate sensor *s*’s missed detection, $c_{k}^{j_{1}j_{2}\cdots j_{S}}$ is the cost of associating the S-tuple of measurements $Z_{k}^{j_{1}j_{2}\cdots j_{S}}$ to a target, and $\rho_{k}^{j_{1}j_{2}\cdots j_{S}}$ is a binary decision variable such that the following is the case.

(7) $$\rho_{k}^{j_{1}j_{2}\cdots j_{S}}=\left\{\begin{array}{cc} 1 & \;\mathrm{if}\;Z_{k}^{j_{1}j_{2}\cdots j_{S}}\;\mathrm{is}\;\mathrm{associated}\;\mathrm{with}\;a\;\mathrm{candidate}\;\mathrm{target}\\ 0 & \;\mathrm{otherwise}\; \end{array}\right..$$

The equality constraints in Equation (6) are to ensure that each measurement is associated with a unique target, or declared false, and that each target is assigned to at most one measurement from each sensor. In Equation (5), cost $c_{k}^{j_{1}j_{2}\cdots j_{S}}$ is defined as the following negative log-likelihood ratio:

(8) $$c_{k}^{j_{1}j_{2}\cdots j_{S}}=-\ln\frac{p\left(Z_{k}^{j_{1}j_{2}\cdots j_{s}}|p_{k}^{i}\right)}{p\left(Z_{k}^{j_{1}j_{2}\cdots j_{s}}|p_{k}^{i}=⌀\right)},$$

where $p\left(Z_{k}^{j_{1}j_{2}\cdots j_{S}}|p_{k}^{i}=⌀\right)$ is the likelihood that measurements in S-tuple $Z_{k}^{j_{1}j_{2}\cdots j_{S}}$ are all spurious, and $p\left(Z_{k}^{j_{1}j_{2}\cdots j_{s}}|p_{k}^{i}\right)$ is the likelihood that these measurements originate from a common target at position $p_{k}^{i}={\left[\xi_{k}^{i}\;\eta_{k}^{i}\;\right]}^{\prime }$. They can be calculated as follows, respectively:

(9) $$p\left(Z_{k}^{j_{1}j_{2}\cdots j_{S}}|p_{k}^{i}=⌀\right)=\prod_{s=1}^{S}{\left[\frac{1}{\psi_{s}}\right]}^{u\left(j_{s}\right)},$$

(10) $$p\left(Z_{k}^{j_{1}j_{2}\cdots j_{S}}|p_{k}^{i}\right)=\prod_{s=1}^{S}{\left(1-P_{D_{s}}\right)}^{1-u\left(j_{s}\right)}{\left[P_{D_{s}}p\left(z_{k}^{s,j_{s}}|p_{k}^{i}\right)\right]}^{u\left(j_{s}\right)},$$

where $\psi_{s}$ is the volume of the field of view of sensor *s*, and $u\left(j_{s}\right)$ is a binary indicator function.

(11) $$u\left(j_{s}\right)=\left\{\begin{array}{cc} 1 & \;\mathrm{if}\;j_{s}\neq 0\;(\;\mathrm{an}\;\mathrm{actual}\;\mathrm{measurement}\;\mathrm{of}\;\mathrm{sensor}\;s)\\ 0 & \;\mathrm{if}\;j_{s}=0\;(\;a\;\mathrm{dummy}\;\mathrm{measurement}\;) \end{array}\right..$$

It should be noted that, in Equation (10), $p_{k}^{i}$ is unknown. Therefore, in order to calculate likelihood $p\left(z_{k}^{s,j_{s}}|p_{k}^{i}\right)$, the corresponding $Z_{k}^{j_{1}j_{2}\cdots j_{S}}$ is used to obtain the maximum likelihood estimation (MLE) of the target position.

(12) $${\hat{p}}_{k}^{i}=\arg\max_{p_{k}^{i}}p\left(Z_{k}^{j_{1}j_{2}\cdots j_{S}}|p_{k}^{i}\right)$$

Substituting Equations (9), (10) and (12) into Equation (8), required cost $c_{k}^{j_{1}j_{2}\cdots j_{S}}$ can be calculated. Note that the optimization problem given by Equations (5) and (6) is NP-hard for S≥3. However, a number of efficient methods to obtain sub-optimal solution have been proposed [46,47,48,49].

## 4. A New Coarse Gating Strategy for MDA

The MLE ${\hat{p}}_{k}^{i}$ of the position of potential target in Equation (12) is a nonlinear optimization problem. In this section, a new coarse gating strategy is proposed to eliminate infeasible association hypotheses by comparing the Mahalanobis distance between the current estimate and initial estimate in an iterative process for the MLE of the target position.

Each S-tuple of measurements $Z_{k}^{j_{1}j_{2}\cdots j_{S}}$ can form a corresponding stacked measurement vector denoted by $z_{k}^{j_{1}j_{2}\cdots j_{S}}={\left[z_{k}^{1,j_{1}},z_{k}^{2,j_{2}},\cdots z_{k}^{S,j_{S}}\right]}^{\prime }$. The relationship between the stacked measurement vector and position of the corresponding target can be written as follows:

(13) $$z_{k}^{j_{1}j_{2}\cdots j_{S}}=\left[\begin{array}{c} z_{k}^{1,j_{1}}\\ z_{k}^{2,j_{2}}\\ \vdots \\ z_{k}^{S,j_{S}} \end{array}\right]=\left[\begin{array}{c} h\left(p_{k}^{i},p_{k}^{1}\right)\\ h\left(p_{k}^{i},p_{k}^{2}\right)\\ \vdots \\ h\left(p_{k}^{i},p_{k}^{S}\right) \end{array}\right]+w_{k}=h\left(p_{k}^{i},p_{k}^{s}\right)+w_{k},\;s=1,2,\cdots ,S$$

where $p_{k}^{i}={\left[\xi_{k}^{i}\;\eta_{k}^{i}\right]}^{\prime }$ is the position of target in XY-plane, $p_{k}^{s}={\left[\xi_{k}^{s}\;\eta_{k}^{s}\right]}^{\prime }$ is the position of sensor *s*, and $w_{k}$ is the stacked measurement vector of measurement noises with covariance $R_{k}=\mathrm{diag}\left(\sigma_{1}^{2},\sigma_{2}^{2},\cdots ,\sigma_{S}^{2}\right)$.

The MLE ${\hat{p}}_{k}^{i}$ of the target position can be solved by iteration, and the iterative process can be denoted [50] by the following:

(14) $${\hat{p}}_{k}^{i,l+1}={\hat{p}}_{k}^{i,l}+{\left({\left(J_{k}^{l}\right)}^{\prime }R_{k}^{-1}J_{k}^{l}\right)}^{-1}{\left(J_{k}^{l}\right)}^{\prime }R_{k}^{-1}\left[z_{k}^{j_{1}j_{2}\cdots j_{S}}-h\left({\hat{p}}_{k}^{i,l},p_{k}^{s}\right)\right]$$

where the following is the Jacobian matrix:

(15) $$J_{k}^{l}={\left.\frac{\partial h\left(p_{k}^{i},p_{k}^{s}\right)}{\partial p_{k}^{i}}\right|}_{p_{k}^{i}={\hat{p}}_{k}^{i,l}}$$

and ${\hat{p}}_{k}^{i,l}$ is the position estimation of target after iteration *l*. The initial estimate ${\hat{p}}_{k}^{i,0}$ can be obtained from the intersection of the bearing measurements of any two of all sensors.

The mean square error of final target position estimate can be calculated by the following.

(16) $$R_{k}^{i,l+1}≜E\left[\left({\hat{p}}_{k}^{i,l+1}-p_{k}^{i}\right){\left({\hat{p}}_{k}^{i,l+1}-p_{k}^{i}\right)}^{\prime }\right]={\left({\left(J_{k}^{i,l}\right)}^{\prime }R_{k}^{-1}J_{k}^{i,l}\right)}^{-1}.$$

For stacked measurement vectors formed by incorrect associations, their elements do not originate from common targets. Therefore, in this case, it is irrational to solve the position estimation given in Equation (12). A natural idea is to analyze the differences of different measurement vectors in the iterative process so as to roughly delete some infeasible associations.

In the iteration, the initial position estimate ${\hat{p}}_{k}^{i,0}$ can be obtained from the intersection of any two bearing components of stacked measurement vector. Moreover, the corresponding covariance $R_{k}^{i,0}$ can be computed by Equation (16). Note that the initial estimate $\left({\hat{p}}_{k}^{i,0},R_{k}^{i,0}\right)$ is determined by the measurements of only two sensors, while the estimate $\left({\hat{p}}_{k}^{i,l},R_{k}^{i,l}\right)$ after *l* iterations, l≥1, is determined by the measurements of all sensors together. That is, these two estimates are not generated by the same measurements. If these measurements are not originated from a common target, the position estimate ${\hat{p}}_{k}^{i,l}$ will deviate from the initial estimate ${\hat{p}}_{k}^{i,0}$ in the iterative process. This will easily result in inconsistencies between these two estimates. Here, the inconsistency between two estimates refers to the fact that the difference between their means is greater than what can be expected based on their respective error covariance estimates [51].

**Figure 5:**
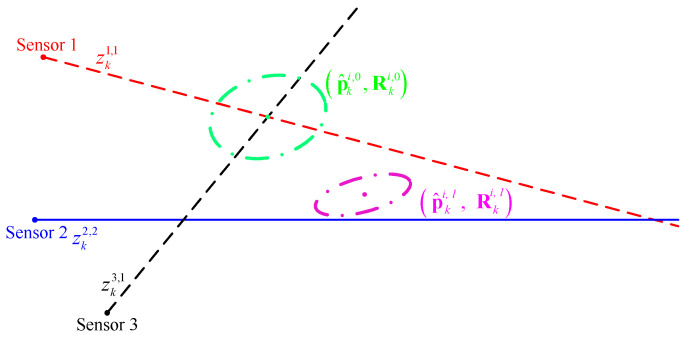
An illustration of the inconsistency between the two estimates. $\left({\hat{p}}_{k}^{i,0},R_{k}^{i,0}\right)$ is the initial estimate and $\left({\hat{p}}_{k}^{i,l},R_{k}^{i,l}\right)$ is the estimate after *l* iterations.

Taking Figure 4c in Section 3 as an example, the stacked measurement vector formed by the S-tuple of measurements $Z_{k}^{121}$ is $z_{k}^{121}={\left[z_{k}^{1,1},z_{k}^{2,2},z_{k}^{3,1}\right]}^{\prime }$. Suppose that, in the iterative process, the initial position estimate $\left({\hat{p}}_{k}^{i,0},R_{k}^{i,0}\right)$ is obtained by the bearing measurements of sensors 1 and 3. If the initial estimate $\left({\hat{p}}_{k}^{i,0},R_{k}^{i,0}\right)$ and the estimate $\left({\hat{p}}_{k}^{i,l},R_{k}^{i,l}\right)$ after *l* iterations, l≥1, are as shown in Figure 5, it means that the two estimates are inconsistent with each other. It should be noted that Figure 5 is only a schematic diagram and not a real experimental result. Numerical experiments will be presented in Section 6.

Therefore, it is necessary to quantitatively analyze the difference between the two estimates. One mechanism for detecting statistically significant deviations between estimates is to calculate the Mahalanobis distance [52]. The Mahalanobis distance between estimates $\left({\hat{p}}_{k}^{i,0},R_{k}^{i,0}\right)$ and $\left({\hat{p}}_{k}^{i,l},R_{k}^{i,l}\right)$ is defined as follows.

(17) $$d_{k}^{i,l}={\left({\hat{p}}_{k}^{i,0}-{\hat{p}}_{k}^{i,l}\right)}^{\prime }{\left(R_{k}^{i,0}+R_{k}^{i,l}\right)}^{-1}\left({\hat{p}}_{k}^{i,0}-{\hat{p}}_{k}^{i,l}\right).$$

It can be roughly interpreted to mean that ${\hat{p}}_{k}^{i,l}$ lies within an ellipsoid centered around ${\hat{p}}_{k}^{i,0}$ [53]. A larger Mahalanobis distance tends to indicate that the two estimates are inconsistent; that is, the components in the corresponding stacked measurement vector do not originate from the common target [51]. Therefore, it is necessary to set an appropriate threshold *T* according to the measurement accuracy of the sensors. When $d_{k}^{i,l}\leq T$, it means that the components may originate from the common target. In this case, iteration (14) will be repeated until $l>N_{\max}$ or the following occurs:

(18) $$\Delta p\;≜\;∥{\hat{p}}_{k}^{i,l+1}-p_{k}^{i,l}∥\;<\;\varepsilon$$

where $N_{\max}$ is preset maximum number of iterations, ∥·∥ is the norm of a vector, ε is a sufficiently small positive real number. Final position estimate ${\hat{p}}_{k}^{i,l}$ will be used to calculate assignment cost $c_{k}^{j_{1}j_{2}\cdots j_{S}}$. When $d_{k}^{i,l}>T$, this means that measurements in the vector originate from different targets. Therefore, the iteration will be terminated and the corresponding association cost will be assigned to infinity.

A threshold *T* is required to detect inconsistencies between the two estimates $\left({\hat{p}}_{k}^{i,0},R_{k}^{i,0}\right)$ and $\left({\hat{p}}_{k}^{i,l},R_{k}^{i,l}\right)$, l≥1, which decides whether it is necessary to further calculate the association cost $c_{k}^{j_{1}j_{2}\cdots j_{S}}$ for MDA. The choice of the threshold *T* is inherently problem dependent [54]. In bearings-only MSMTT, it is closely related to the position and measurement accuracy of the passive sensors. In order to avoid deleting incorrect associations, the threshold should not be too small. For a small number of remaining incorrect associations, the subsequent MDA can be used for further identification. In practical applications, an a priori threshold can be determined in advance with the help of cooperative targets.

For some infeasible associations, terminating the iterations when the Mahalanobis distance between the initial estimate of the iterative estimate is greater than a set threshold *T* can effectively save computational cost. The proposed strategy is denoted by coarse gating in iterations (CGI). The CGI-driven MDA is summarized in Algorithm 1.
**Algorithm 1:** CGI driven MDA
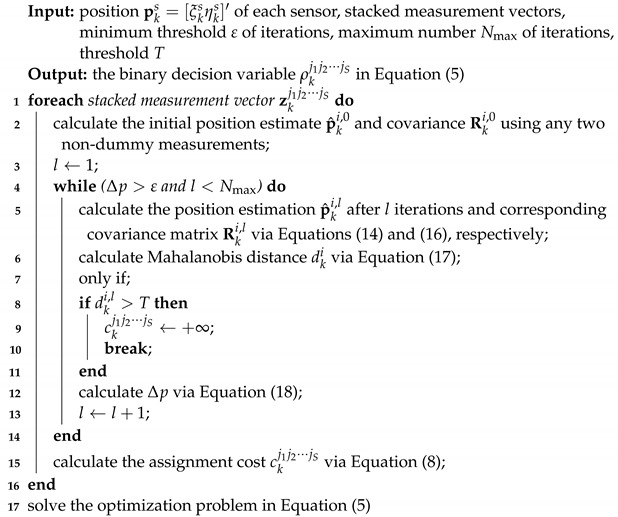


## 5. Two-Stage MSMTT

**Figure 6:**
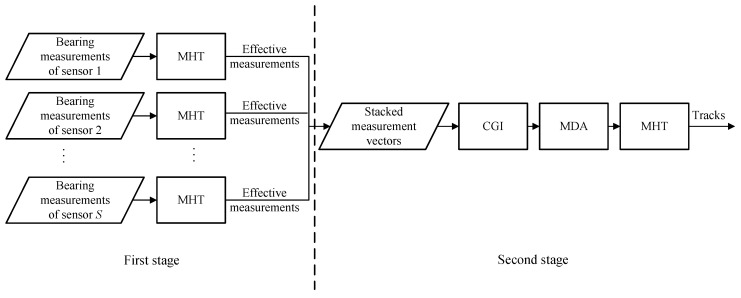
Framework of TS-MHT.

In this section, the CGI-driven MDA is combined with a TS-MHT framework to perform bearings-only MSMTT. The framework is given in Figure 6.

First, MHT is performed at each sensor, and only the measurements used to update the tracks are sent to the FC. Here, these measurements are referred to as “effective measurements.” Second, the effective measurements from different sensors are combined and augmented to form stacked measurement vectors. Note that each measurement vector is a potential association hypothesis. The proposed CGI is then used to eliminate infeasible association hypotheses. After this, the measurement-to-measurement association is performed using the MDA algorithm. Finally, target tracks are obtained by using the second stage MHT.

The advantages of the above framework are mainly in the following aspects. In the framework shown in Figure 6, using the first stage MHT can eliminate most of the false measurements obtained by individual sensors, thus reducing the number of stacked measurement vectors. This further reduces the computational requirement of associations, and it also helps improve the accuracy of MDA. In turn, accurate data association facilitates track initialization in the second stage MHT and avoids infeasible hypothesis generation.

### 5.1. First Stage MHT

For the first stage, bearings-only multitarget tracking needs to be performed at each local passive sensor. Many existing methods are available [23,33,55]. Since this part is not the focus of this work, only one of the methods is considered.

The method proposed in [33] is to define the target state in Cartesian coordinates, thus performing single sensor state-space tracking. It should be noted that in [33], the target moves in three-dimensional space, and frequency information is available. In order to use the strategy for two-dimensional bearings-only MSMTT, it is simplified so that the dynamical system of the target can be described by Equations (1) and (2).

First, the one-point initialization approach is performed by combining the detection range of the sensor and all measurements at the initial time. Suppose that the detection range of sensor *s* is within the interval $\left[r_{\min}^{s},r_{\max}^{s}\right]$. Correspondingly, the initial range between the target and the sensor and the corresponding variance can be calculated [33] as follows.

(19) $$r^{s}=\frac{r_{\min}^{s}+r_{\max}^{s}}{2},\;\sigma_{r}^{2}=\frac{{\left(r_{\max}^{s}-r_{\min}^{s}\right)}^{2}}{12}.$$

Then, the estimate of the initial state vector and the associated covariance are the following:

(20) $${\hat{x}}_{0|0}^{j_{s}}=\left[\begin{array}{c} x_{0}^{j_{s}}\\ {\dot{x}}_{0}^{j_{s}}\\ y_{0}^{j_{s}}\\ {\dot{y}}_{0}^{j_{s}} \end{array}\right]=\left[\begin{array}{c} r^{s}\sin\left(z_{0}^{s,j_{s}}\right)+x_{0}^{s}\\ 0\\ r^{s}\cos\left(z_{0}^{s,j_{s}}\right)+y_{0}^{s}\\ 0 \end{array}\right],$$

(21) $$P_{0|0}^{j_{s}}=J^{\prime }RJ,$$

where the following is the case:

(22) $$R=\mathrm{diag}\left(\sigma_{r}^{2},\sigma_{s}^{2},\sigma_{\dot{x}}^{2},\sigma_{\dot{y}}^{2}\right),$$

(23) $$J=\frac{\partial z_{0}^{s,j_{s}}}{\partial {\hat{x}}_{0|0}^{j_{s}}},$$

and $\sigma_{s}^{2}$ represents the measurement noise variance of sensor *s*, and $\sigma_{\dot{x}}^{2}$ and $\sigma_{\dot{y}}^{2}$ are the velocity variances based on their a priori maximum values.

It should be noted that, for this method, parameter $r^{s}$ is only used for track initiation. That is to say that only bearing measurements are used to update tracks during the course of track maintenance. In addition, the measurements used for updating will be sent to the second stage.

### 5.2. Second Stage MHT

After the first stage MHT, most false measurements from each local sensor are eliminated, and the effective measurements are sent to the FC. Considering that the tracking performance of single passive sensor is quite limited in the first stage, these effective measurements can be divided into three categories: measurements originated from the target, false measurements due to false association, and dummy measurements due to missed detection. Therefore, in the second stage, the measurement-to-measurement association still needs to be performed.

First, all effective measurements from different sensors are combined and augmented to form stacked measurement vectors. Each stacked measurement vector is a potential association hypothesis. Then, the proposed CGI is used to delete infeasible associations. For each stacked measurement vector, in the iterative process of obtaining the MLE of target position, if the Mahalanobis distance $d_{k}^{i,l}$ between the initial estimate $\left({\hat{p}}_{k}^{i,0},R_{k}^{i,0}\right)$ and the iterative estimate $\left({\hat{p}}_{k}^{i,l},R_{k}^{i,l}\right)$ is greater than threshold *T*, then the association is determined as infeasible and deleted. When $d_{k}^{i,l}\leq T$, the estimate from the final iteration is naturally regarded as the MLE of the target position in the XY-plane, i.e., the solution of Equation (12). At the same time, it can be used for subsequent MDA. Finally, target tracks are obtained through the second stage MHT.

## 6. Illustrative Examples

In this section, five illustrative examples are presented. First, a scenario with three stationary targets (Scenario 1) is used to illustrate that, for incorrect associations, the initial estimation and iterative estimation generated in the iterative process are often inconsistent so as to verify the rationality and feasibility of the proposed strategy CGI. Second, a scenario with 18 stationary targets (Scenario 2) is used to compare the performance difference of three methods, MDA, CGI-driven MDA, and clustering-based MDA [43], to verify the effectiveness of the proposed strategy. Finally, a single-target tracking scenario (scenario 3) and multi-target tracking scenarios (scenarios 4 and 5) are used to further validate the performance of the framework shown in Figure 6.

### 6.1. Verification of Inconsistency

This subsection uses a numerical example about stationary targets to illustrate the difference in Mahalanobis distance between the current and initial estimates in an iterative process for the MLE of different target positions so as to verify the feasibility of the CGI proposed in Section 4.

Suppose there are three fixed passive sensors located at (0 m, 0 m), (1000 m, 600 m), and (3000 m, 0 m) in the XY-plane. At time *k*, sensor s,s∈{1,2,3}, acquires bearing measurements $\left\{z_{k}^{s,1},z_{k}^{s,2}\right\}$, where $z_{k}^{s,1}$ and $z_{k}^{s,2}$ represents the measurements originated from the targets 1 and 2, respectively. The positions of these two targets in the XY-plane are (1500 m, 200 m) and (1800 m, 500 m). The standard deviations of the measurement errors of these three sensors are $\sigma_{s}=17.5$ mrad, s∈{1,2,3}.

**Figure 7:**
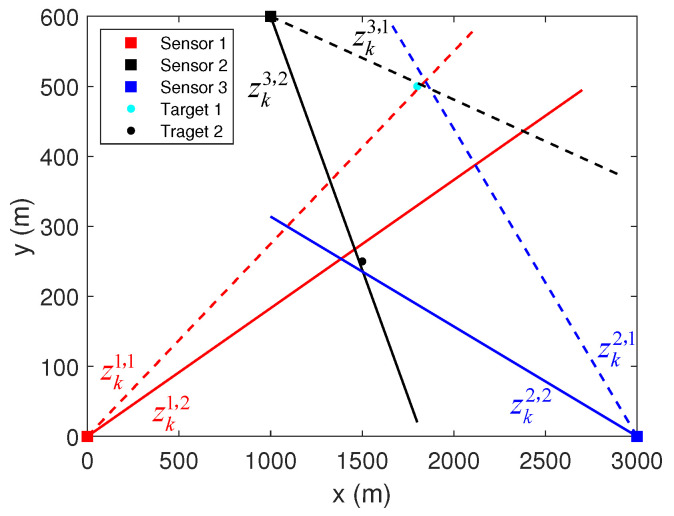
Scenario 1 with 2 stationary targets and 3 passive sensors.

**Figure 8:**
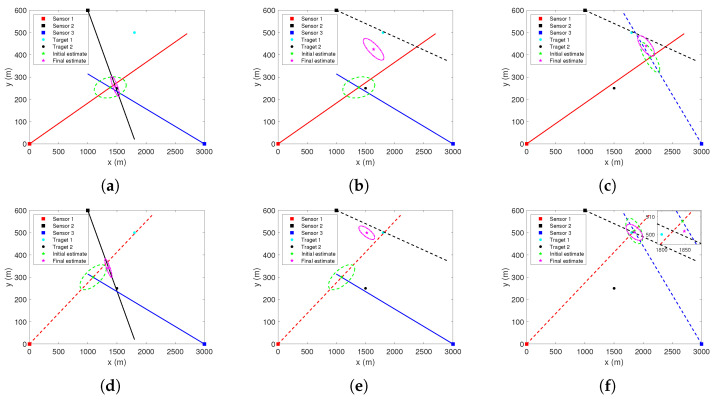
The initial estimate and the estimate after *l* iterations obtained using different stacked measurement vectors. (**a**) $z_{k}^{111}$. (**b**) $z_{k}^{121}$. (**c**) $z_{k}^{122}$. (**d**) $z_{k}^{211}$. (**e**) $z_{k}^{221}$. (**f**) $z_{k}^{222}$.

**Figure 9:**
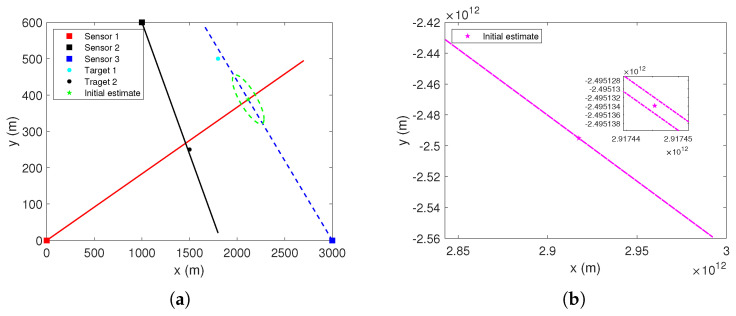
The initial estimate and the estimate after *l* iterations obtained using the stacked measurement vector $z_{k}^{112}$. (**a**) Initial estimate. (**b**) Estimate after *l* iterations.

**Figure 10:**
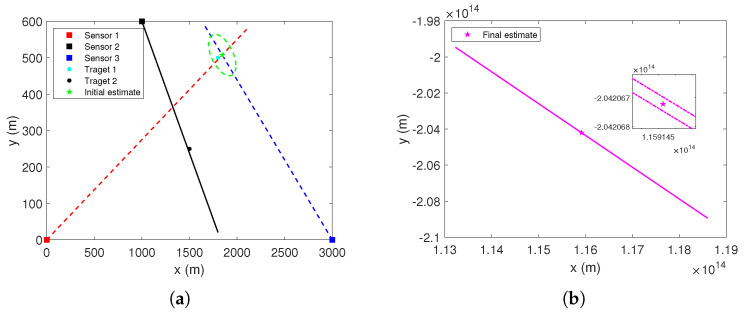
The initial estimate and the estimate after *l* iterations obtained using the stacked measurement vector $z_{k}^{212}$. (**a**) Initial estimate. (**b**) Estimate after *l* iterations.

In the absence of false alarms and missed detections, eight stacked measurement vectors, i.e., association hypotheses, can be obtained. Figure 7 shows the bearing measurements of each sensor in one of the Monte Carlo runs, where the dashed lines represent the measurements originated from target 1, and the solid lines represent the measurements originated from target 2. Figure 8, Figure 9 and Figure 10 show initial estimate $\left({\hat{p}}_{k}^{i,0},R_{k}^{i,0}\right)$ and iterative estimate $\left({\hat{p}}_{k}^{i,l},R_{k}^{i,l}\right)$, $l=N_{\max}$ obtained using these stacked measurement vectors. Note that the only condition for iteration termination in this scenario is $l>N_{max}$. The uncertainty of the position estimates in the XY-plane is represented by the 95% probability ellipses.

From Figure 8a,f, when all components of the stacked measurement vector originate from the same target, the uncertainty ellipse of the iterative estimate is smaller than that of the initial estimate, and these two estimates are consistent. From Figure 8b,d,e, it can be observed that these two estimates obtained by $z_{k}^{121}$, $z_{k}^{211}$, and $z_{k}^{221}$ are inconsistent. For the other two stacked measurement vectors $z_{k}^{112}$ and $z_{k}^{212}$, since the initial and iterative estimates are too far away from each other, they are shown in the subfigures of Figure 9 and Figure 10, respectively. It can be observed that the uncertainty ellipses of the iterative estimates are extremely large. For this two cases, the initial and iterative estimates are also obviously inconsistent.

It can be demonstrated through the above experiments that for many infeasible associations, the two estimates, $\left({\hat{p}}_{k}^{i,0},R_{k}^{i,0}\right)$ and $\left({\hat{p}}_{k}^{i,l},R_{k}^{i,l}\right)$, obtained in the iterations are often inconsistent.

Furthermore, for each stacked measurement vector, the Mahalanobis distances between initial estimate $\left({\hat{p}}_{k}^{i,0},R_{k}^{i,0}\right)$ and all iterative estimates $\left({\hat{p}}_{k}^{i,l},R_{k}^{i,l}\right)$, $l=\{1,2,\cdots ,N_{\max}\}$ are calculated. Table 1 presents the minimum and maximum Mahalanobis distances obtained in the iterative process with the different stacked measurement vectors. It is the statistic obtained from 2000 Monte Carlo runs.

It can be observed that, throughout the iterative process, the Mahalanobis distances obtained by using correctly associated vectors $z_{k}^{111}$ and $z_{k}^{222}$ are significantly smaller. Therefore, infeasible associations can be effectively eliminated by setting an appropriate threshold *T*.

### 6.2. CGI Driven MDA for Stationary Targets

**Figure 11:**
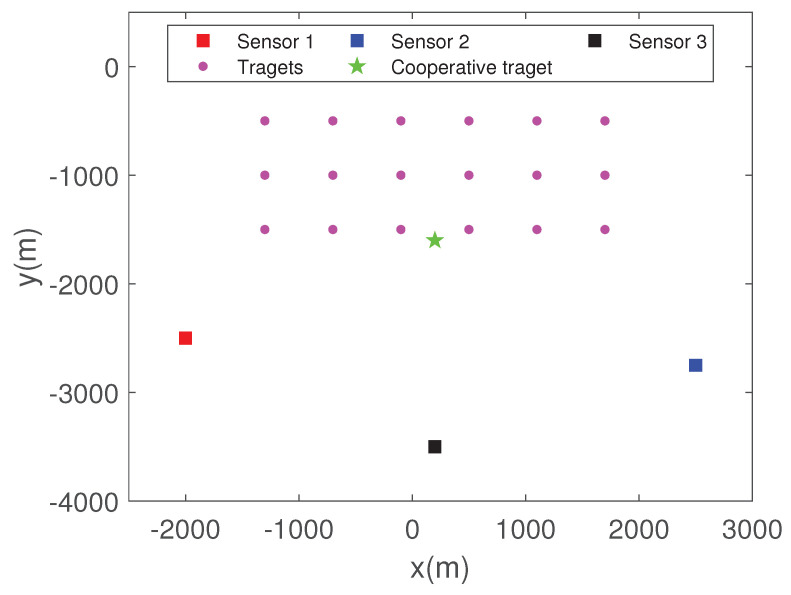
Scenario 2 with 18 stationary targets and 3 passive sensors.

In this subsection, the impact of the proposed CGI on the performance of MDA will be analyzed. This scenario, as illustrated in Figure 11, consists of 3 bearing-only passive sensors, 1 cooperative target, and 18 unknown non-cooperative targets.

The positions of three passive sensors in the XY-plane are (−2000 m, −2500 m), (2500 m, −2750 m), and (200 m, −3500 m), respectively. The standard deviations of the measurement errors for all sensors are $\sigma_{s}=1$ mrad, s∈{1,2,3}. The position of the cooperative target is (600 m, −1600 m). The positions of other unknown non-cooperative targets are shown in Table 2. For the sake of simplicity, it is assumed that all sensors have unity detection probability for each target and there are no false measurements. It is also supposed that, at some point before these unknown targets are detected, three passive sensors can only acquire the bearing measurements originating from the cooperative target.

In order to set a reasonable threshold *T* for the proposed CGI, the bearing measurements originating from the cooperative target are used iteration of Equations (14) and (16). The maximum Mahalanobis distance between the initial estimate $\left({\hat{p}}_{k}^{i,0},R_{k}^{i,0}\right)$ and each iterative estimate $\left({\hat{p}}_{k}^{i,l},R_{k}^{i,l}\right)$, $l\in \{1,2,\cdots N_{\max}\}$ was $d_{\max}=11.6977$ over 2000 Monte Carlo runs. Considering that the Mahalanobis distance is closely related to the geometric structure between the sensors and the cooperative target, threshold *T* should not be less than $d_{\max}$. In order to avoid deleting the correct association, the threshold in this scenario is set to T=12.

The Lagrangian relaxation method in [48] is used to obtain suboptimal solutions of the MDA problem in Equation (5). Table 3 presents the performance comparison of three different methods, MDA, CGI driven MDA, and clustering-based MDA [43], based on a 2000-run Monte Carlo average. The experimental results are obtained on MATLAB R2020b with Intel(R) Core(TM) i5-9500 CPU @3.00GHz and RAM of 8 GB.

It can be observed from Table 3 that the number of S-tuples reduced from 5832 to 83.82 after CGI. Moreover, the correct association rate of CGI-driven MDA is 99.61%, much higher than that of the other two methods. This means that CGI can effectively eliminate a large number of infeasible associations and can significantly improve the correct association rate of MDA. In addition, for MDA, the execution time to calculate the assignment costs of all S-tuples is 3.9069 s. This takes about 81% of the total execution time. For CGI-driven MDA, the execution time for calculating all assignment costs is only 3 s, which accounts for about 50% of the total execution time. Obviously, the proposed CGI driven MDA has a significant improvement in both computational efficiency and correct association probability. For the clustering-based MDA method, the execution time of obtaining the suboptimal solution of Equation (5) by the Lagrangian relaxation algorithm is less than that of the proposed CGI-driven MDA method. This is due to the fact that the clustering method decomposes the entire assignment problem into smaller subproblems, thus improving computational efficiency.

Table 4 illustrates the impact of five different thresholds on the performance of the proposed CGI-driven MDA. It can be observed that the larger *T* is, the larger the number of S-tuples obtained after CGI, and the more execution time is required. The correct association rate when T=1 is 78.14%, which is less than the correct association rate when T=12. Therefore, it is not the case that the smaller the threshold is, the better. Smaller thresholds may result in the removal of some correct associations. Moreover, it can be found that when T=24, the correct association rate is significantly smaller than the other two groups. This is mainly due to the large number of retained S-tuples, which results in the degradation of the performance of the Lagrangian relaxation algorithm used.

### 6.3. TS-MHT for Single Target Tracking in Clutter

In this subsection, a single target tracking scenario is considered to verify the performance of the TS-MHT framework shown in Figure 6.

**Figure 12:**
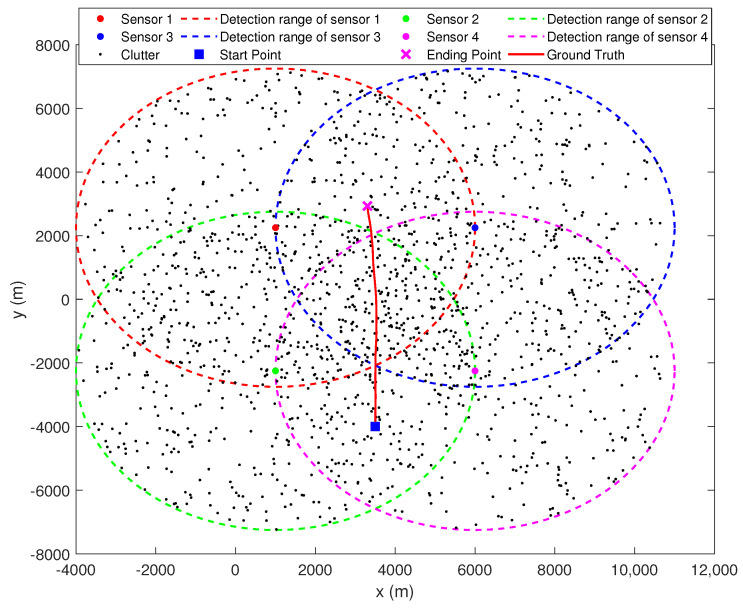
Scenario 3 for single target tracking with 4 passive sensors.

There are four passive sensors located at (1000 m, 2250 m), (1000 m, −2250 m), (6000 m, 2250 m), and (6000 m, −2250 m), respectively. Each sensor can only measure the bearing to the target, and the sampling interval is 10 s. Their measurement errors are modeled as zero-mean Gaussian white noises with same standard deviations $\sigma_{s}=17.5$ mrad, s∈{1,2,3,4}. The maximum detection range of each sensor is 5 km and the detection probability is $P_{D_{s}}=0.9$, s∈{1,2,3,4}. False measurements are uniformly distributed over the detection range and their number is Poisson distributed with an average of 4 false measurements per sensor per scan. Target moves in two dimensions with NCV, and its initial position and velocity are [3500 m, −4000 m] and [0 m/s, 7.2 m/s], respectively. The process noise covariance is $Q=0.01^{2}I$. The true trajectory of the target motion and the sensor positions are shown in Figure 12, where the detection range of each sensor is indicated by dashed lines of different colors.

**Figure 13:**
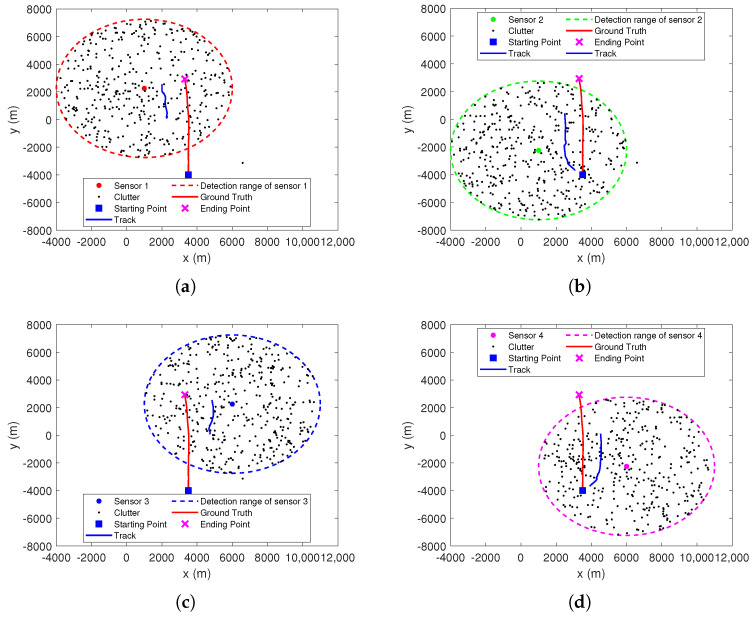
Tracking results of first stage MHT in each local sensor. (**a**) Sensor 1. (**b**) Sensor 2. (**c**) Sensor 3. (**d**) Sensor 4.

Figure 13 shows the tracking results of each sensor at the first stage. Here, threshold *T* is set to 16. Superficially, there are significant differences between the tracking results of each sensor and the true track of the target. This is due to the unobservability of target state for single passive sensor. Although, in track initiation, the initial position estimate of the target can be obtained by the detection range of the sensor, it is also inaccurate.

It should be noted that, for the first stage of the TS-MHT framework shown in Figure 6, its main purpose is to eliminate as many false measurements as possible by using preliminary tracking. Moreover, only the measurements used to update these tracks are sent to the second stage in real time. That is to say that it is more interested in whether the measurements sent to the second stage originate from the true target than in the accuracy of target state estimation. From Figure 13, it can be observed that the number of tracks obtained by sensors are all one. These estimated numbers of tracks are close to the number of true target. In addition, the estimated tracks by each sensor and the true track of the target are on the same side of the corresponding sensor, and their orientations with respect to the sensors are roughly the same. This means that the tracking results of the first stage may not be that bad, although the tracking results still need to be further improved by the measurements from other sensors during the second stage.

**Figure 14:**
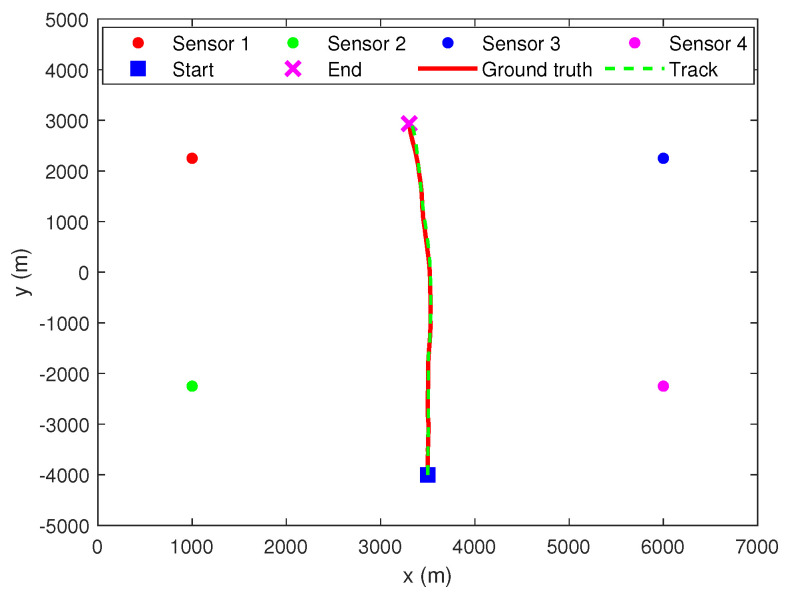
Tracking results of second stage MHT.

Figure 14 shows the tracking result of the second stage. It can be observed that the second stage MHT can effectively track the target in clutter. At the same time, this in turn shows that CGI-driven MDA can effectively delete infeasible associations.

The execution time of each stage is calculated over 2000 Monte Carlo runs. For the first stage, the average execution time per frame of the MHT algorithm in each sensor is approximately equal, and it is about 1.0741 s. For the second stage, the average execution time of MHT algorithm is 0.1782 s per frame. Obviously, the execution time of the second stage MHT is significantly smaller than that of the first stage MHT. This is due to the fact that the first stage can effectively eliminate a large number of false measurements, thus effectively reducing the number of feasible assumptions in the second stage. It should be noted that the effective measurements in the first stage are sent to the second stage in real time.

**Figure 15:**
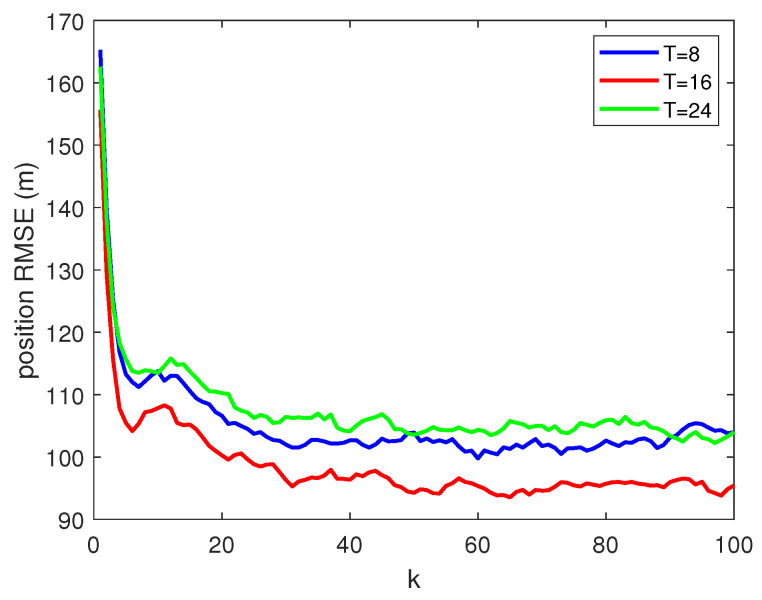
Position RMSE of different threshold *T*.

In addition, to verify the effect of different thresholds *T* on tracking performance, the root mean square error (RMSE) is used to measure the performance of target tracking, as shown in Figure 15. It can be observed that when T=16, tracking performance is significantly better than the other two groups. Combined with the experimental results of Scenario 2, it can be further demonstrated that when preset threshold *T* is too large or too small, and it may result in a decrease in the correct association rate of MDA, which further affects tracking performance.

### 6.4. TS-MHT for Multitarget Tracking in Clutter

**Figure 16:**
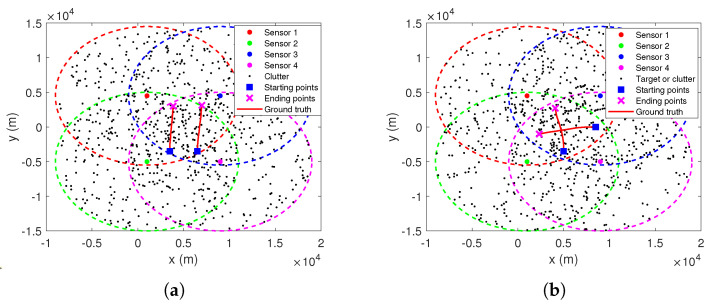
Multitarget tracking scenarios with 4 passive sensors. (**a**) Scenarios 4. (**b**) Scenarios 5.

Consider two multitarget tracking scenarios with four sensors. For scenario 4, as shown in Figure 16a, the two targets move simultaneously along the *Y* direction with a nearly constant speed of 6.2 m/s, and their initial positions are [3500 m, −3500 m] and [6500 m, −3500 m], respectively. For scenario 5 as shown in Figure 16b, the initial positions of the two targets are [5000 m, −3500 m] and [8500 m, 0 m], and their initial velocities are [0 m/s, 7.2 m/s] and [−5.2 m/s, 0 m/s], respectively. The other parameters are the same as in Scenario 2.

**Figure 17:**
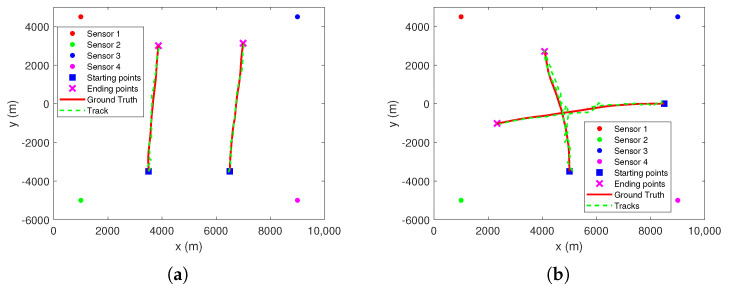
Tracking results. (**a**) Scenarios 4. (**b**) Scenarios 5.

By comparing Figure 16 and Figure 17, it can be observed that the proposed strategy can effectively tackle MSMTT.

## 7. Conclusions

The bearings-only multitarget tracking problem is investigated for synchronous passive sensors. In the target tracking process, especially for track initiation, MDA can be used to identify the measurements originating from common targets. In order to reduce the computational cost of the multidimensional assignment and improve its correct association rate, a new coarse gating strategy, the CGI, has been proposed first. For MDA, iterative processes can be used to obtain the MLE of target position corresponding to each possible association and, thus, further calculate the assignment cost of that association. Since the initial estimate and the iterative estimate are not obtained by the same measurements, it has been proposed to eliminate infeasible associations by using the Mahalanobis distance between the initial estimate and the iterative estimate as a measure. The feasibility and effectiveness of the proposed CGI is verified by two scenarios, i.e., scenarios 1 and 2, respectively. In addition, MDA driven by this strategy is combined with the TS-MHT framework for distributed MSMTT. Numerical examples have verified the performance of the proposed strategy. Moreover, the effectiveness of the proposed strategy in the tracking process is further verified by two scenarios of single target and multitarget in clutter.

## Figures and Tables

**Table 1:** Mahalanobis distances between the initial estimate and the iterative estimates.

	$z_{k}^{111}$	$z_{k}^{112}$	$z_{k}^{121}$	$z_{k}^{122}$	$z_{k}^{211}$	$z_{k}^{212}$	$z_{k}^{221}$	$z_{k}^{222}$
Minimum Mahalanobis distances	0.8336	80.9022	17.9394	3.8289	4.7382	102.5766	15.8057	0.4970
Maximum Mahalanobis distances	1.1744	$5.1467\times 10^{4}$	45.7706	4.7942	7.0418	$2.1429\times 10^{4}$	56.0292	0.5112

**Table 2:** Positions of all targets in *XY*-plane.

(−1500 m, −500 m)	(−900 m, −500 m)	(−300 m, −500 m)	(900 m, −500 m)	(1500 m, −500 m)	(−1500 m, −500 m)
(−1500 m, −1000 m)	(−900 m, −1000 m)	(−300 m, −1000 m)	(900 m, −1000 m)	(1500 m, −1000 m)	(−1500 m, −1000 m)
(−1500 m, −1500 m)	(−900 m, −1500 m)	(−300 m, −1500 m)	(900 m, −1500 m)	(1500 m, −1500 m)	(−1500 m, −1500 m)

**Table 3:** The performance comparison of different methods.

	MDA	Clustering-Based MDA	CGI Driven MDA
Number of all S-tuples	5832	5832	5832
Number of S-tuples after coarse gating	-	103.74	83.82
Number of identified targets	19.58	18.97	18.02
Percent correct association	33.35%	81.67%	99.61%
Execution time to calculate assignment costs	3.9069 s	0.3917 s	0.3625 s
Execution time to obtain suboptimal solution	0.9163 s	0.1457 s	0.3543 s

**Table 4:** The effect of different threshold *T* on the performance of CGI driven MDA.

	T=1	T=6	T=12	T=16	T=24
Number of all S-tuples	5832	5832	5832	5832	5832
Number of S-tuples after CGI	29.43	61.03	83.82	95.89	114.93
Number of identified targets	18.27	18.01	18.02	18.37	19.68
Percent correct association	78.14%	99.33%	99.61%	87.61%	69.39%
Execution time to calculate assignment costs	0.3597	0.3397 s	0.3625 s	0.3472 s	0.3439 s
Execution time to obtain suboptimal solution	0.0164	0.0964 s	0.3543 s	0.8761 s	1.3270 s

## Data Availability

Not applicable.

## Abbreviations

The following abbreviations are used in this manuscript:

MSMTT	Multisensor-multitarget tracking;
MDA	Multidimensional assignment;
MLE	Maximum likelihood estimation;
TS-MHT	Two-stage multiple hypothesis tracking;
MTT	Multitarget tracking;
GNN	Global nearest neighbor;
PDA	Probabilistic data association;
JPDA	Joint probabilistic data association;
JIPDA	Joint integrated probabilistic data association;
MS-JPDA	Multiscan joint probabilistic data association;
MHT	Multiple hypothesis tracking;
RFS	Random finite set;
PHD	Probability hypothesis density;
CPHD	Cardinalized probability hypothesis density;
GLMB	Generalized labeled multi-Bernoulli;
FC	Fusion center;
CGI	Coarse gating in iterations;
2D	Two-dimensional;
RMSE	Root mean square error;
NCV	Nearly constant velocity.

## Nomenclatures

**Notations**	**Definitions**
*S*	Number of sensors
*s*	Sensor index, s=1,2,⋯,S
*i*	Target index
*k*	Time index
$j_{s}$	Bearing measurement index acquired by sensor *s*
$N_{s}$	Number of bearing measurements acquired by sensor *s*
$z_{k}^{s,j_{s}}$	The $j_{s}$ th bearing measurement acquired by sensor *s* at time *k*
$\beta_{k}^{s,i_{s}}$	True bearing between target *i* and sensor *s*
${\tilde{z}}_{k}^{j_{s}}$	False measurements
$x_{k}^{i}$	State vector of target *i* at time *k*
$p_{k}^{s}$	Position vector of sensor *s*
$F_{k}$	State transition matrix at time *k*
$w_{k}^{i}$	Process noise vector of target *i* at time *k*
$v_{k}^{s,j_{s}}$	Measurement noise of sensor *s* at time *k*
$\sigma_{s}^{2}$	Measurement noise variance of sensor *s*
$Z_{k}^{j_{1}j_{2}\cdots j_{S}}$	An S-tuple of measurements, one from each sensor
$c_{k}^{j_{1}j_{2}\cdots j_{S}}$	Cost of associating S-tuple of measurements with a target
$\rho_{k}^{j_{1}j_{2}\cdots j_{S}}$	Binary decision variables
$P_{D_{s}}$	Detection probability of sensor *s*
$\psi_{s}$	Volume of the field of view of sensor *s*
$u\left(j_{s}\right)$	Binary indicator function
*l*	iteration index
$z_{k}^{j_{1}j_{2}\cdots j_{S}}$	Stacked measurement vector corresponding to S-tuple $Z_{k}^{j_{1}j_{2}\cdots j_{S}}$
$p_{k}^{i}$	Position vector of target *i*
${\hat{p}}_{k}^{i,0}$	Initial position estimate of target *i*
${\hat{p}}_{k}^{i,l}$	Position estimation of target *i* after iteration *l*
$R_{k}^{i,l}$	Covariance matrix corresponding to the position estimate ${\hat{p}}_{k}^{i,l}$
$J_{k}^{l}$	Jacobian matrix
$d_{k}^{i,l}$	Mahalanobis distance
*T*	Threshold
